# Seasonality of associations between production of indigenous foods and food security status of mother-child dyads in Kisumu County, Kenya

**DOI:** 10.1186/s40795-024-00820-6

**Published:** 2024-01-10

**Authors:** Kenneth Kipngeno Tonui, Agatha Christine Onyango, Collins Ouma

**Affiliations:** 1 School of Public Health and Community Development, Department of Nutrition and Health, Maseno University, 333-40105 Maseno, Kenya; 2 School of Public Health and Community Development, Department of Biomedical Sciences and Technology, Maseno University, 333-40105 Maseno, Kenya

**Keywords:** Indigenous foods, Food security status, Mothers and children, Food sufficiency, Kisumu county

## Abstract

**Background:**

Food insecurity is a major predicament for rural populations, especially mothers and children, whose livelihoods are often dependent on rain-fed agriculture. Indigenous foods have the potential of mitigating food insecurity as they can thrive in poor agro-ecological conditions. However, the associations between indigenous food production and food security status of mothers and children drawn from rural contexts has not been expansively assessed. Food insecurity evident by high food poverty rates remain high in Kisumu County due to over-reliance on food imports from other counties. The objective of the study was to assess seasonality in associations between production of selected indigenous foods (kidney beans, soya beans, millet, cassava, sweet potatoes, groundnuts, green grams, cow peas, amaranth leaves, spider plant leaves, black night shade leaves, mangoes, guavas, lime, and tamarind) and food security status of mothers and children during planting and harvesting seasons.

**Methods:**

We used a longitudinal study design adopting both quantitative and qualitative data collection methods. A structured questionnaire assessed production of selected indigenous foods in the sampled households, Household Food Insecurity Access Scale for mother’s food security status and Prevalence of Underweight for children’s food security status. Ordinal logistic regression was used to derive odds ratio (OR), which assessed strength of associations between dependent variables (mother’s and children’s food security status) and independent variables (production of selected indigenous foods). Significance was determined at α ≤ 0.05.

**Results:**

Results demonstrated that during planting season, production of kidney beans decreased the odds of mothers being severely food insecure by 53% (OR = 0.469, 95% CI = 0.228–0.964, *p* = 0.039). In the same season, sorghum production demonstrated 3.5 times increase in odds of children being severely food insecure (OR = 3.498, 95% CI = 1.454–8.418, *p* = 0.005). During harvesting season, production of kidney beans was associated with a 62% reduction in the odds of children being severely food insecure (OR = 0.379, 95% CI = 0.190–0.754, *p* = 0.006).

**Conclusions:**

Production of some of the selected indigenous foods demonstrated significant odds of predicting mother’s and children’s food security status across both study seasons. An intervention-based study approach that would best establish causal associations of indigenous food production and food security status is recommended.

## Background

The concept of food security has undergone varying conceptual shifts since its emergence during the mid-1970s. At that time, food security was conceptualized in regards to food supply/availability for the growing global population [1]. As such, food security was defined based on global food supply, with no reference to food supply at national and local contexts. In the late 1970s and early 1980s the concept was down-scaled to depict food sufficiency at national/country levels. Initial concepts on food security laid more focus on food quantity aspect with little regard for food quality component [1]. The 1980s experienced a shift in food security concepts where emphasis was laid on access component, which led to the eventual conceptualization of food security as a household issue rather than a solely national matter [2]. It is as a result of the historical shifts that led to current conceptualization of four food security components; food availability, access, utilization, and stability.

Food security exists in situations whereby the populations, specifically households, have guaranteed physical, social, and economic access to food that is enough and of good quality [3], which satisfies individual’s dietary preference and needs; hence, enable them to lead a healthy and fulfilled lifestyle [4]. Thus, food insecurity refers to situations where populations do not have sustained access to enough food that is of adequate quality; hence, cannot meet their dietary preferences and daily needs to enable them to lead a healthy lifestyle [5]. On the other hand, indigenous foods refer to plant and animal-based foods that are natively produced or naturally occur in particular locations; hence, they differ depending on respective geographical locations [6]. For this paper, we focused on specific indigenous foods including; kidney beans, millet, soya beans, sorghum, sweet potatoes, cassava, groundnuts, green grams, cow peas, amaranth leaves, black night shade leaves, spider plant leaves, mangoes, guavas, lime, and tamarind.

The UNICEF conceptual framework on causes of undernutrition underscores the colossal role that food security plays in mitigating undernutrition, which poses adverse nutrition and health implications [7]. The framework identifies household food insecurity as an underlying cause of maternal and child undernutrition as it causes inadequate dietary intake; intake of diets low in quantity and quality, which leads to undernutrition [8]. Deductively, undernutrition that may be attributable to food insecurity, results in short-term and long-term health consequences. Some of the short-term consequences include disability, morbidity, and mortality, whereas, long-term consequences, which are often inter-generational include impaired cognitive ability, impaired reproductive performance, reduced economic productivity, metabolic and cardiovascular diseases, and stunting [7].

Sustainable agriculture has the potential to address immediate and long-term needs of populations predisposed to food insecurity [9, 10]. Agricultural food production, which is the backbone of many rural livelihoods, provides a means of attaining resilience to food insecurity [11]. Indigenous food production provides a comprehensive basis for mitigating food insecurity crisis, especially for rural livelihoods who are dependent on agricultural food production [12]. Production through cultivation or gathering from their natural habitat is an indicator of indigenous food production at the household level [13].

Global food insecurity continues to deteriorate due to worldwide economic slowdowns, which impair sustainable food access by the populations [14]. This situation is exacerbated by climate change that has resulted in unpredictable rain patterns; hence, impairing adequate food crop production [15]. The COVID-19 pandemic further worsened the situation and made it challenging to attain Sustainable Development Goal (SDG) number 2, which envisions to end hunger by the year 2030 [14, 16]. This was evident by close to 118 million more people experiencing food insecurity in 2020 than in 2019 [16]. Projections on lasting effects of COVID-19 show that close to 660 million will be food insecure in 2030, which is 30 million more than the initial projections in the event that the pandemic hand not occurred [16].

Globally, women and children are disproportionately affected by food insecurity whereby two-thirds of 828 million people affected by food insecurity in 2021 were women [17, 18]. It is further estimated that gender gap in food security amongst women and men has increased 8.4 times since 2018 [18]. Prevalence of Undernourishment (PoU) indicates food security status amongst children whereby in 2020, 149.2 million (22.0%) children were stunted, 45.4 million (6.7%) were wasted, and 38.9 million (5.7%) were overweight at the global level [16]. Undernourishment of children is a direct result of food insecurity [19]. Asia and Sub-Saharan Africa (SSA) bear a huge burden of food insecurity crisis whereby in 2020, of all the people affected by food insecurity, there were 57 million more from Asia and 46 million more from Africa [16].

In Kenya, food security situations remain dire, especially for populations drawn from counties such as the study area; Kisumu County, that majorly rely on food imports from neighbouring counties [20]. Climate change that has resulted in failed/short rains for the last two years coupled with high food prices has rendered close to 4 million Kenyans food insecure [21], while in Kisumu, food poverty rate is 61% [22]. Food insecurity poses dire consequences such as adoption of socially unacceptable ways of acquiring food, household members experience hunger due to inability to afford food, household members worrying that food will run out before they get more supply, and consumption of non-nutritious and less diverse diets by affected households [12, 14]. Local food production provides a viable and comprehensive platform that support sustained food security in any given context [20].

A high proportion of populations drawn from low-income contexts with poor agro-ecological conditions such as poor soil conditions and limited rainfall live an ever-increasing vulnerability to nutrition and food insecurity, as well as hunger. This is the case in Seme Sub-County, a rural sub-county situated along the shorelines of Lake Victoria in Kenya. The area has and continues to experience high food insecurity levels evidenced by statistics indicating 26.3% of children aged below five years are stunted. Furthermore, a paltry 17.6% of children aged 6–23 months drawn from the study area consume the recommended minimum meal frequency with food insecurity being a major hindrance to adoption of appropriate breastfeeding practices [23].

Existing strategies aimed at improving food and nutrition systems often focus on “new world foods,” or commercially expensive food crops, which require high level of financial investments [23]. Such investments are not feasible, especially for the rural poor such as those of Seme Sub-County, Kisumu County, Kenya who have limited resource capabilities. Profitable crop production in the study area is further limited by poor soil texture (sandy soil) coupled with its marginal suitability to rain-fed agriculture [12]. Indigenous foods have a potential of thriving in poor resource settings with harsh agro-ecological conditions such as Seme Sub-County; hence, can help mitigate food insecurity [13]. Thus, this paper provides guidelines on the potential value of indigenous food in the development and implementation of workable approaches aimed at enhancing food security in the study area. It further provides a clear understanding of food security status in Seme Sub-County, Kisumu County, Kenya, which is fundamental for the development of policies and standards aimed at enhancing food security in the study location and regions with similar features. Besides, the paper provides practical evidence in the budding body of literature of possible role of indigenous foods in enhancing food security status.

## Methods

The study adopted a longitudinal study design using a mixed methods approach, whereby qualitative data complemented the quantitative data. Data was collected in two study seasons; season I (planting season) and season II (harvesting season) in Seme Sub-County, Kisumu County, Kenya. The area is more predisposed to food insecurity as it relies on food imports from other counties and due to its marginal dependence on rain-fed agriculture [22]. A sample of 193 Households computed using Creative Research Methods (2003) formula, with at least one mother aged 18 to 49 years with a singleton child aged 12 to 36 months who had resided in the study area for at least two years were included. The two-year reference period was to ensure that sampled households gave a true reflection of the study variables; production of selected indigenous foods and food security status of mothers and children, in the study context. Quantitative data were collected using a structured questionnaire yielding information on demographic features of sampled households, indigenous food production practice, Household Food Insecurity Access Scale (HFIAS), and Prevalence of Undernourishment (PoU). Qualitative data were collected using Key Informant Interviews (KIIs) and Focus Group Discussions (FGDs) probing understandings and opinions on food security and indigenous foods.

The corresponding author conducted FGD sessions with seven [7] male and five [5] female household heads who were purposively recruited. The researcher held an initial meeting with the FGD participants prior the FGD sessions whereby the research objectives were clearly explained and consent to audio record their responses sought. The study separately conducted one face-to-face FGD session among the female and male FGD participants. Each FGD was carried out at a location accessible, agreed upon by the FGD participants, and lasted for one and a half hours. The study developed KI and FGD interview guides that were pre-tested and administered during the FGD and KII sessions. The researcher facilitated and moderated the FGD sessions, but played a passive role; hence, allowed participants to discuss their views with little or no influence. While moderating the sessions, the researcher observed for saturation at a time when similar responses to interview prompts were received from most of the participants. For KII’s the study purposively recruited six [6] Key Informants (KIs); Seme sub-county nutrition coordinator, sub-county rural development officer, a program manager (nutrition programming) working with a Non-Governmental Organization; International Centre for AIDS Care and Treatment Programs, a community leader (mother’s support group chairperson), rural development officer, and assistant director public health department. The questionnaire used for quantitative and qualitative data collection for this study was adopted from a Kenyan study that explored agricultural food production and household food security status [23]. Responses from the FGDs and KIIs were audio-recorded with permission from the participants.

Dependent variable was categorical and comprised information on food security status measured using Food and Nutrition Technical Assistance (FANTA) 2013 HFIAS for mothers and PoU for children. Mother’s food security status was based on HFIAS prevalence categorized as food secure, mildly food insecure access, moderately food insecure access, and severely food insecure access. For children, PoU measures were equated to three food security status categories; food secure (normal weight-for-age z-score; ≥ -2), moderately food insecure (moderate malnourished; < -2 to ≥ -3 weight-for-age z-score), and severely food insecure (weight-for-age z-score; < − 3). PoU was used as a measure children’s food security status since it is a composite measure of chronic and acute undernutrition, majorly attributable to food insecurity [24]. Independent variable was production/cultivation of selected indigenous foods by the participant’s households.

Data was analyzed using descriptive and inferential statistics. Descriptive statistics, mainly frequencies, mean, and percentages were used. For inferential statistics, Pearson Chi-square test was used to determine the proportionality of dependent and independent variables. Ordinal logistic regression with PLUM procedure was used to determine the odds of independent variables (production of selected indigenous foods) predicting the occurrence of dependent variables (food security status of mothers and children. Significance was determined at α ≤ 0.05 (95% confidence level).

Qualitative data were analysed using content analysis whereby transcription of responses from FGDs and KIIs for emergent themes following a model of analytical induction was done. The study formulated themes and patterns of analysis for establishment of thematic structures. Emerging themes from qualitative data analysis were organized into coherent categories as a means of explaining specific components of the study findings.

## Results

The study targeted 193 mother-child dyads; however, 189 mother-child dyads were accessible (response rate: 97.9%). Table 1 displays the sociodemographic characteristics of the sampled participant’s households. The mean maternal age was 27.68 years (± 6.40 SD). The age of the mothers varied from 18 to 44 years whereby a majority; 88 (46.6%) belonged to the age category of 18 to 24 years. In terms of marital status, a majority; 158 (83.6%) of the participants were married and slightly more than average; 106 (56.1%) were of Pentecostal religion. Almost three-quarters; 133 (70.4%) of the participants were drawn from households comprising of 3 to 6 household members with the mean household size being 5.63 (± 1.86 SD). Almost all the participants; 179 (94.7%) reported that the mother was not the head of the household. Slightly more than half; 102 (54.0%) of the children were female. Furthermore, the study found that the mean age in years for the children was 1.94 (± 0.64 SD). More than three-quarters, 150 (77.7%) of the participants reported that they had completed primary school level of education.

**Table 1:** Sociodemographic characteristics of sampled households

Characteristic	***n***	Percentage (%)
**Age Group (Years)**		
18–24	88	46.6
25–31	57	30.2
32–38	32	16.9
39–44 Mean age 27.68 (± 6.40 SD)	12	6.3
**Marital Status**		
Married	158	83.6
Never Married	20	10.6
Separated	2	1.1
Divorced	1	0.5
Widowed	8	4.2
**Religion**		
Catholic	28	14.8
Anglican	19	10.1
SDA	9	4.8
Pentecostal	106	56.1
Legion Maria	5	2.6
Muslim	22	11.6
**Household Size**		
3–6	133	70.4
7–10	53	28.0
11–14 Mean Household Size 5.63 (± 1.86 SD)	3	1.6
**Mother Household Head?**		
Yes	10	5.3
No	179	94.7
**Education level completed**		
None	8	4.1
Primary	150	77.7
Secondary	33	17.1
Tertiary	2	1.0

*n* = number of participants (*n* = 189)

Table 2 displays the profile of participants recruited for the Focused Group Discussions (FGDs) and Key Informant Interviews (KIIs). For the male FGDs, all (100%) reported to have completed secondary school level of education. A high proportion, 4 (57.1%) of them were aged 45 to 49 years. For the female FGDs, more than half, 3 (60.0%) reported to have completed secondary schools level of education and most of them, 4 (80%) were aged 40 to 45 years. For the KIIs, a high percentage, 4 (66.7%) had completed post-graduate level of education, and an average, 3 (50.0%) were aged 35 to 39 years. In terms of gender, most of the KIIs, 4 (66.7%) were females.

**Table 2:** Profile of FGD and KII participants

Characteristic	***n***	Percentage (%)
**Male FGD participants**		
**Age**		
30–34	1	14.3
35–39	1	14.3
40–44	1	14.3
45–49	4	57.1
**Education level completed**		
Secondary	7	100
**Female FGD participants**		
**Age**		
35–39	1	20.0
40–44	4	80.0
**Education level completed**		
Primary	2	40.0
Secondary	3	60.0
**KII participants**		
**Age**		
30–34	1	16.7
35–39	3	50.0
40–44	1	16.7
45–49	1	16.7
**Education level completed**		
Undergraduate	2	33.3
Post-graduate	4	66.7
**Gender**		
Males	2	33.3
Females	4	66.7

*n* = number of participants: Male FGDS (*n* = 7), female FGD (*n* = 7), KIIs (*n* = 6)

Result (Table 3) shows indigenous food production practice by the participant’s households. Slightly above average production was reported for mangoes only as produced by 97(51.3%) of the households. Production of the other selected indigenous foods was below average. The mean land ownership was 1.14 (± 3.69 SD) acres. The mean size of land cultivated by the recruited households was 1.03 (± 1.67 SD) acres.

**Table 3:** Indigenous food production practices in the participants households

Proportion of participants producing selected indigenous foods		
Indigenous food	*n*	Percentage (%)
Kidney beans	71	37.6
Soya beans	5	2.6
Millet	7	3.7
Sorghum	34	18.0
Cassava	57	30.2
Sweet potatoes	52	27.5
Groundnuts	70	37.0
Green grams	19	10.1
Cow peas	53	28.0
Amaranth leaves	54	28.6
Spider plant leaves	59	31.2
Black nightshade leaves	30	15.9
Mangoes	97	51.3
Guavas	79	41.8
Lime	42	22.2
Tamarind	10	5.3

Mean Land Ownership: **1.14 (± 3.69 SD) Acres**

Mean Size of Land Cultivated: **1.03 (± 1.67 SD) Acres**

*n* = number of participants (*n* = 189)

Information from the Focused Group Discussions (FGDs) and Key Informant Interviews (KIIs) corroborated the quantitative information regarding indigenous food production by the participant’s households. Table 4 provides a summary of qualitative data derived from content analysis. A high proportion of the FGD and KII participants reported that production of indigenous food is not fully adequate because they did not produce in all seasons.

*“….the farms are small and we have accepted to transition from traditional foods into modern day foods because we have been made to believe that they have good nutrition value and fetch more profits when taken for sale in the market”* (FGD_1_, 2022).

*“……there is little focus on home-grown solutions to food insecurity in the community such as introduction of traditional foods and edible insects, which are draught resistant. Such strategies would offer sustainable solutions to food insecurity”* (KII_6_, 2022).

**Table 4:** Summary of results from content analysis

Theme	Description of relevant themes	Relevant quotes
Running out of food or worrying about food running out	Participants generally mentioned that often at times they run out of food or are worried that they will run out of food.	“In many instances, I am worried that I may not be able to provide food for my family. At times, I even go away from home, until am able to guarantee that there will be food for my household” (Male FGD, 2022). “Providing sufficient food for our households on a daily basis has become a challenge since most for the times we depend on casual jobs, “*vibarua*,” which are hard to come by these days” (Female FGD, 2022).
Frequency of occurrence of worry or running out of food	Participants observed that there are specific times when they run out of food or worry that food will not be enough for the family. This was mainly dependent on type of livelihood the participants engaged in. For those in salaried employment, the third and fourth weeks were the critical times when there was worry of running out of food. For those in contractual/casual employment, worry was more apparent when they were not engaged in any contract or casual labour.	“Not having a stable job (salaried employment) means that I will only have sustained food access when I get contractual jobs, which are not guaranteed” (Male FGD, 2022). “Not so many times do we run out or worry about running out of food, it happens once in a while because we have foods such as cassava, which save the situation most of the times” (Female FGD, 2022).
Coping strategies/options	Participants reported adopting different coping strategies in the event that they lack food such as reducing size of meals, eating cheaper foods, watering down ingredients, selling household assets, and borrowing.	“We eat what we never used to eat before and at times you abscond your duty as a provider and leave the dependents to borrow from neighbours or survive from the little that is available in the kitchen garden” (Male FGD, 2022). “We serve children first, and serve less food to yourself, but more for the father so that he gets energy to go and look for more food for next days” (Female FGD, 2022).
Indigenous food production	Most of the participants reported that they produce different types of indigenous foods, but production was not fully adequate: not available in all seasons.	“We have accepted the transition to “modern day foods” because we have been made to believe that they have good nutrition value and are profitable when sold in the market” (Female FGD, 2022). “The farms are small and end up exhausting nutrients in the soil because they are cultivated every season” (Male FGD, 2022).
Food security status of the community and evidence thereof	Most of the KII’s reported that there is a general lack of sustained access to safe, sufficient, and healthy food by the community members.	“Sustained access to food in sufficient quantity and of adequate quality is not guaranteed for a significant proportion of the rural community households. This is because there are limited livelihood opportunities in the rural set up when compared to urban set ups” (KII_3_, 2022). “A common example of evidence of limited food access is the consumption of less-preferred (unacceptable) such as *mumi and akoko* (type of fishes), which are not acceptable amongst Adventists and Nomiya religious adherents” (KII_2_, 2022).
Women and child nutrition issues within the community	KII’s observed that maternal and infant child issues have not been accorded the attention it deserves and players in various sectors including the national and county governments, as well as the non-governmental sector should take lead in advocating and empowering the community of these issues	“Different existing dynamics points to little consideration of mother and child issues. As an example, a daughter-in-law is not allowed to bring harvest to the household in the absence of the mother-in-law. Such affect intra-household food distribution: hence, rendering women and other family members, especially children vulnerable to food insecurity” (KII_4_, 2022).

**Fig. 1:**
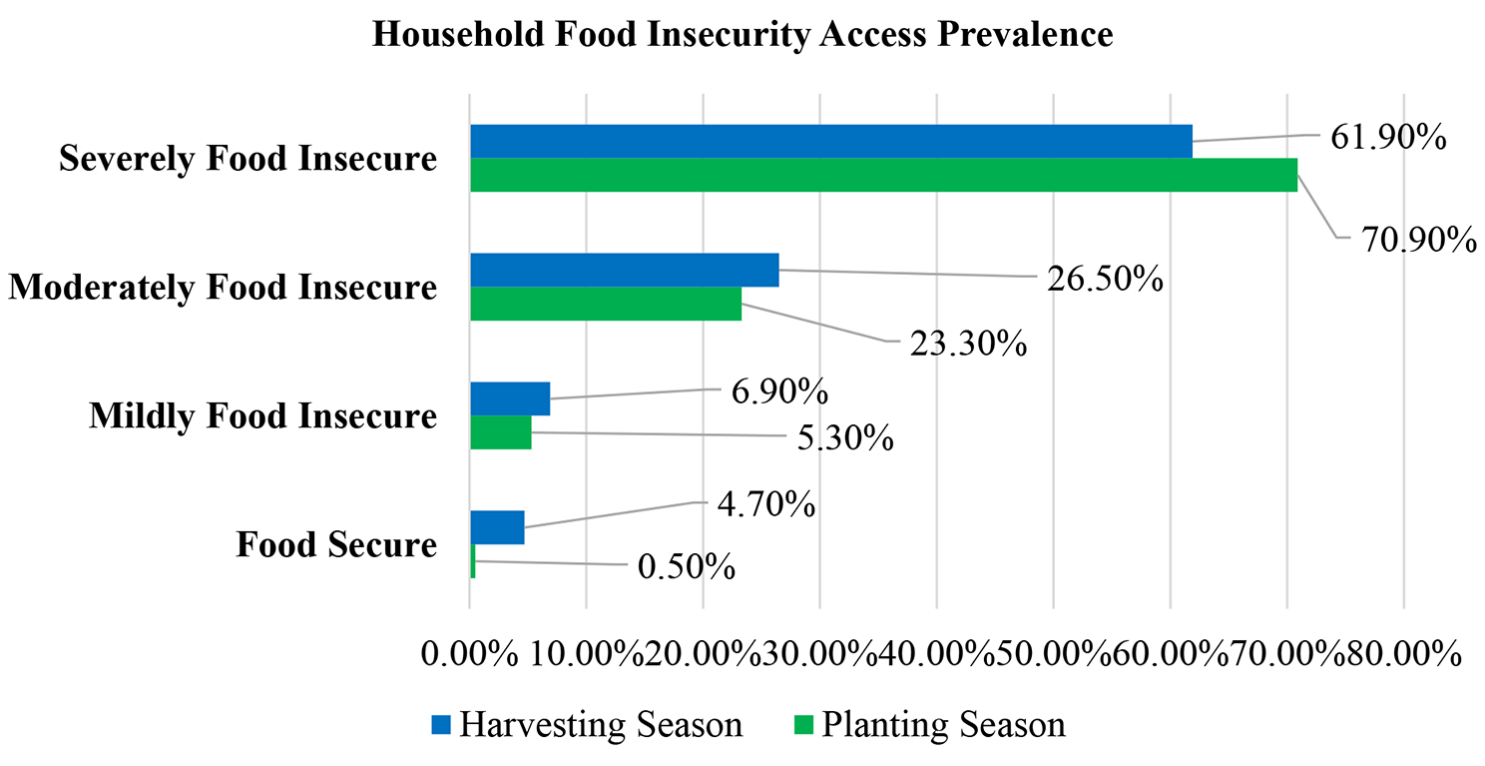
Mothers food security status during planting and harvesting seasons

In planting season, a majority of the mothers; 134 (70.9%) experienced severe food insecurity prevalence (Fig. 1). This was also the case during harvesting season whereby a majority of the mothers; 117 (61.9%) experienced severe food insecurity prevalence. FGD participants expressed that that food insecurity was a core concern amongst the study population whereby in many instances where households run out of food or are worried that they will run out of food. The frequency of occurrence of such instances were varying depending on the stability of livelihood engagement of the household members.

*“……many at times, I am worried that I may not be in a position to provide food for my households. In fact, there are times I go away from the homestead until such a time when I am in a position for provide food for them”* (FGD_2_, 2022).

*“…….providing food sufficiently on a daily basis is quite a challenge because a high number of households depend on contractual jobs; “vibarua,” which are hard to come by these days”* (FGD_1_, 2022).

The mean weight-for-age Z score for planting season: -0.69 (± 1.06 SD) was statistically significantly higher than the mean weight-for-age Z score for harvesting season: -1.18 (± 1.28 SD) at α ≤ 0.05 (t-test, *p* < 0.0001). In planting season, almost three-quarters of the children; 137 (72.5%) were food secure. On the other hand, slightly more than half; 102 (54.0%) of the children were food secure during harvesting season (Table 5).

**Table 5:** Food security status of children segregated by planting and harvesting seasons

Food Security Status	Planting Season		Harvesting Season	
	*n*	%	*n*	%
Food Secure	137	72.5	102	54.0
Moderate Food Insecurity	46	24.3	59	31.2
Severe Food Insecurity	6	3.2	28	14.8

*n* is the number of child participants (*n* = 189). PoU measures were equated to three food security status categories; food secure (normal weight-for-age z-score; ≥ -2), moderately food insecure (moderate malnourished; < -2 to ≥ -3 weight-for-age z-score), and severely food insecure (weight-for-age z-score; < − 3)

This was consistent with participant’s view that coping strategies adopted by households affected by food insecurity were likely to compromise the overall food and nutrition security of the children.

“…….*household heads, especially fathers are served first so that they get energy to go and look for food for coming days. Children may be served more and women less and last”* (FGD_1_ & FGD_2_, 2022).

As shown in Table 6, during planting season, production of kidney beans decreased the odds of mothers being severely food insecure by 53% (OR = 0.469, 95% CI = 0.228–0.964, *p* = 0.039). Mother’s food security status was not associated with production of soya beans (*p* = 0.233), millet (*p* = 0.496), sorghum (*p* = 0.183), cassava (*p* = 0.592), sweet potatoes (*p* = 0.567), ground nuts (*p* = 0.475), green grams (*p* = 0.226), cow peas (*p* = 0.556), amaranth (*p* = 0.583) spider plant (*p* = 0.640), black night shade (*p* = 0.782), mangoes (*p* = 0.426), guavas (*p* = 0.669), lime (*p* = 0.852), and tamarind (*p* = 0.340).

**Table 6:** Associations between indigenous food production and mother's food security status during planting season

Variables	Food Security Status				*χ* ^2^	*p*-value	OR	95% CI	*p*-value
	Food secure *n* (%)	Mildly food insecure *n* (%)	Moderately food insecure *n* (%)	Severely food insecure *n* (%)					
**Indigenous food production**									
Kidney Beans									
Yes	1 (0.5)	5 (2.6)	23 (12.2)	42 (22.2)	8.591	0.035*	0.469	0.228–0.964	0.039*
No	0 (0.0)	5 (2.6)	21 (11.1)	92 (48.7)					
Soya Beans									
Yes	0 (0.0)	0 (0.0)	3 (1.6)	2 (1.0)	3.965	0.265	0.285	0.036–2.242	0.233
No	1 (0.5)	10 (5.3)	41 (21.7)	132 (69.8)					
Millet									
Yes	0 (0.0)	1 (0.5)	1 (0.5)	5 (2.6)	1.403	0.705	1.991	0.274–14.480	0.496
No	1 (0.5)	9 (4.8)	43 (22.8)	129 (68.3)					
Sorghum									
Yes	0 (0.0)	4 (2.1)	9 (4.8)	21 (11.1)	4.172	0.243	0.552	0.231–1.324	0.183
No	1 (0.5)	6 (3.2)	35 (18.5)	113 (59.8)					
Cassava									
Yes	1 (0.5)	3 (1.6)	17 (9.0)	36 (19.0)	4.507	0.212	0.796	0.345–1.834	0.592
No	0 (0.0)	7 (3.7)	27 (14.3)	98 (51.9)					
Sweet Potatoes									
Yes	1 (0.5)	3 (1.6)	15 (7.9)	33 (17.5)	4.180	0.243	0.797	0.366–1.735	0.567
No	0 (0.0)	7 (3.7)	29 (15.3)	101 (53.4)					
Ground Nuts									
Yes	1 (0.5)	3 (1.6)	22 (11.6)	44 (23.3)	6.097	0.107	0.761	0.359–1.610	0.475
No	0 (0.0)	7 (3.7)	22 (11.6)	90 (47.6)					
Green Grams									
Yes	0 (0.0)	0 (0.0)	4 (2.1)	15 (7.9)	1.467	0.690	2.445	0.576–10.386	0.226
No	1 (0.5)	10 (5.2)	40 (21.2)	119 (63.0)					
Cow Peas									
Yes	0 (0.0)	2 (1.0)	17 (9.0)	34 (18.0)	3.631	0.304	0.796	0.373–1.701	0.556
No	1 (0.5)	8 (4.2)	27 (14.3)	100 (52.9)					
Amaranth leaves									
Yes	0 (0.0)	3 (1.6)	13 (6.9)	38 (20.1)	0.433	0.933	1.254	0.559–2.810	0.583
No	1 (0.5)	7 (3.7)	31 (16.4)	96 (50.8)					
Spider Plant									
Yes	0 (0.0)	3 (1.6)	14 (7.4)	42 (22.2)	0.469	0.926	1.225	0.524–2.863	0.640
No	1 (0.5)	7 (3.7)	30 (15.9)	92 (48.7					
Black nightshade									
Yes	0 (0.0)	1 (0.5)	10 (5.2)	19 (10.1)	2.283	0.516	0.868	0.319–2.363	0.782
No	1 (0.5)	9 (4.8)	34 (18.0)	115 (60.9)					
Mangoes									
Yes	1 (0.5)	4 (2.1)	28 (14.8)	64 (33.9)	4.812	0.186	0.720	0.320–1.619	0.426
No	0 (0.0)	6 (3.2)	16 (8.5)	70 (37.0)					
Guavas									
Yes	1 (0.5)	4 (2.1)	22 (11.6)	52 (27.5)	3.116	0.374	1.192	0.532–2.669	0.669
No	0 (0.0)	6 (3.2)	22 (11.6)	82 (43.4)					
Lime									
Yes	0 (0.0)	1 (0.5)	12 (6.3)	29 (15.3)	1.825	0.609	0.917	0.370–2.273	0.852
No	1 (0.5)	9 (4.8)	32 (16.9)	105 (55.6)					
Tamarind									
Yes	0 (0.0)	0 (0.0)	3 (1.6)	7 (3.7)	0.821	0.845	2.175	0.440-10.742	0.340
No	1 (0.5)	10 (5.2)	41 (21.7)	127 (67.2)					

*Pearson Chi-Square (χ2) test for associations. *Statistically significant χ2 association at α ≤ 0.05. OR odds ratio, 95% CI: 95% Confidence Interval. OR generated through ordinary logistic regress using PLUM test procedure. Classifications on food (in) security status were derived based on the frequency of occurrence of food insecurity experiences and respective coping strategies adopted

During harvesting season, mother’s food security status was not comparable to production of kidney beans (*p* = 0.934), soya beans (*p* = 0.552), millet (*p* = 0.812), sorghum (*p* = 0.097), cassava (*p* = 0.232), sweet potatoes (*p* = 0.087), ground nuts (*p* = 0.509), green grams (*p* = 0.837), cow peas (*p* = 0.442), amaranth (*p* = 0.809), spider plant (*p* = 0.053), black night shade (*p* = 0.152), mangoes (*p* = 0.222), guavas (*p* = 0.178), lime (*p* = 0.381), and tamarind (*p* = 0.382) (Table 7).

**Table 7:** Associations between indigenous food production and mother's food security status during harvesting season

Variables	Food Security Status				*χ* ^2^	*p*-value	OR	95% CI	*p*-value
	Food secure	Mildly food insecure	Moderately food insecure	Severely food insecure					
	*n* (%)	*n* (%)	*n* (%)	*n* (%)					
**Indigenous food production**									
Kidney Beans									
Yes	4 (2.1)	4 (2.1)	19 (10.1)	44 (23.3)	0.442	0.931	1.029	0.523–2.025	0.934
No	5 (2.6)	9 (4.8)	31 (16.4)	73 (38.6)					
Soya Beans									
Yes	1 (0.5)	1 (0.5)	0 (0.0)	3 (1.6)	5.152	0.161	0.550	0.077–3.933	0.552
No	8 (4.2)	12 (6.3)	50 (26.5)	114 (60.3)					
Millet									
Yes	1 (0.5)	0 (0.0)	1 (0.5)	5 (2.6)	2.398	0.494	1.241	0.208–7.397	0.812
No	8 (4.2)	13 (6.9)	49 (25.9)	112 (59.3)					
Sorghum									
Yes	2 (1.0)	3 (1.6)	10 (5.2)	19 (10.1)	0.717	0.869	0.492	0.213–1.136	0.097
No	7 (3.7)	10 (5.2)	40 (21.2)	98 (51.9)					
Cassava									
Yes	1 (0.5)	4 (2.1)	16 (8.5)	36 (19.0)	1.654	0.647	1.635	0.729–3.667	0.232
No	8 (4.2)	9 (4.8)	34 (18.0)	81 (42.9)					
Sweet Potatoes									
Yes	5 (2.6)	3 (1.6)	15 (8.0)	29 (15.3)	4.268	0.234	0.527	0.253–1.097	0.087
No	4 (2.1)	10 (5.2)	35 (18.5)	88 (46.6)					
Ground Nuts									
Yes	4 (2.1)	4 (2.1)	15 (8.0)	47 (24.9)	1.985	0.575	1.272	0.623–2.598	0.509
No	5 (2.6)	9 (4.8)	35 (18.5)	70 (37.0)					
Green Grams									
Yes	0 (0.0)	3 (1.6)	4 (2.1)	12 (6.3)	3.683	0.298	0.886	0.281–2.798	0.837
No	9 (4.8)	10 (5.2)	46 (24.3)	105 (55.6)					
Cow Peas									
Yes	3 (1.6)	3 (1.6)	11 (5.8)	36 (19.1)	1.620	0.655	1.329	0.643–2.749	0.442
No	6 (3.2)	10 (5.2)	39 (20.6)	81 (42.9)					
Amaranth leaves									
Yes	1 (0.5)	4 (2.1)	14 (7.4)	35 (18.5)	1.487	0.685	1.099	0.514–2.350	0.809
No	8 (4.2)	9 (4.8)	36 (19.1)	82 (43.4)					
Spider Plant									
Yes	0 (0.0)	3 (1.6)	15 (8.0)	41 (21.7)	5.318	0.150	2.239	0.990–5.065	0.053
No	9 (4.8)	10 (5.2)	35 (18.5)	76 (40.2)					
Black nightshade									
Yes	0 (0.0)	2 (1.0)	11 (5.8)	17 (9.0)	3.264	0.353	0.497	0.191–1.294	0.152
No	9 (4.8)	11 (5.8)	39 (20.6)	100 (52.9)					
Mangoes									
Yes	3 (1.6)	5 (2.6)	27 (14.3)	62 (32.8)	2.300	0.512	1.595	0.754–3.370	0.222
No	6 (3.2)	8 (4.2)	23 (12.2)	55 (29.1)					
Guavas									
Yes	6 (3.2)	6 (3.2)	20 (10.6)	47 (24.8)	2.583	0.460	0.602	0.288–1.260	0.178
No	3 (1.6)	7 (3.7)	30 (15.9)	70 (37.0)					
Lime									
Yes	1 (0.5)	3 (1.6)	13 (6.9)	25 (13.2)	1.111	0.775	0.689	0.300-1.585	0.381
No	8 (4.2)	10 (5.2)	37 (19.6)	92 (48.7)					
Tamarind									
Yes	0 (0.0)	1 (0.5)	2 (1.0)	7 (3.7)	0.930	0.818	1.976	0.429–9.098	0.382
No	9 (4.8)	12 (6.4)	48 (25.4)	110 (58.2)					

*Pearson Chi-Square (χ2) test for associations. *Statistically significant χ2 association at α ≤ 0.05. OR odds ratio, 95% CI: 95% Confidence Interval. OR generated through ordinary logistic regression using PLUM test procedure

As shown in Table 8, during planting season, production of sorghum demonstrated 3.5 times increase in odds of children being severely food insecure (OR = 3.498, 95% CI = 1.454–8.418, *p* = 0.005). However, children’s food security status was not associated with production of; kidney beans (*p* = 0.741), soya beans (*p* = 0.430), millet (*p* = 0.647), cassava (*p* = 0.377), sweet potatoes (*p* = 0.210), ground nuts (*p* = 0.273), green grams (*p* = 0.328), cow peas (*p* = 0.247), amaranth (*p* = 0.644), spider plant (*p* = 0.997), black night shade (*p* = 0.708), mangoes (*p* = 0.671), guavas (*p* = 0.535), lime (*p* = 0.877), and tamarind (*p* = 0.597).

**Table 8:** Associations between indigenous food production and children's food security status during planting season

Variables	Food Security Status			*χ* ^2^	*p*-value	OR	95% CI	*p*-value
	Food secure	Moderately food insecure	Severely food insecure					
	*n* (%)	*n* (%)	*n* (%)					
**Indigenous food production**								
Kidney Beans								
Yes	50 (26.5)	17 (9.0)	4 (2.1)	2.241	0.326	1.136	0.535–2.412	0.741
No	87 (46.0)	29 (15.3)	2 (1.0)					
Soya Beans								
Yes	4 (2.1)	1 (0.5)	0 (0.0)	0243	0.886	0.355	0.027–4.634	0.430
No	133 (70.4)	45 (23.8)	6 (3.2)					
Millet								
Yes	4 (2.1)	3 (1.6)	0 (0.0)	1.491	0.474	1.497	0.267–8.413	0.647
No	133 (70.4)	43 (22.7)	6 (3.2)					
Sorghum								
Yes	19 (10.1)	12 (6.3)	3 (1.6)	7.789	0.020*	3.498	1.454–8.418	0.005*
No	118 (62.4)	34 (18.0)	3 (1.6)					
Cassava								
Yes	42 (22.2)	14 (7.4)	1 (0.5)	0.536	0.765	0.672	0.278–1.624	0.377
No	95 (50.3)	32 (16.9)	5 (2.7)					
Sweet Potatoes								
Yes	40 (21.2)	10 (5.3)	2 (1.0)	1.066	0.587	0.569	0.235–1.374	0.210
No	97 (51.3)	36 (19.0)	4 (2.1)					
Ground Nuts								
Yes	47 (24.9)	21 (11.1)	2 (1.0)	1.937	0.380	1.543	0.710–3.351	0.273
No	90 (47.6)	25 (13.2)	4 (2.1)					
Green Grams								
Yes	12 (6.3)	7 (3.7)	0 (0.0)	2.281	0.320	1.838	0.543–6.220	0.328
No	125 (66.1)	39 (20.6)	6 (3.2)					
Cow Peas								
Yes	39 (20.6)	14 (7.4)	0 (0.0)	2.481	0.289	0.610	0.264–1.408	0.247
No	98 (51.9)	32 (16.9)	6 (3.2)					
Amaranth leaves								
Yes	39 (20.6)	13 (6.9)	2 (1.0)	0.070	0.966	0.826	0.366–1.863	0.644
No	98 (51.9)	33 (17.5)	4 (2.1)					
Spider Plant								
Yes	40 (21.2)	17 (9.0)	2 (1.0)	0.979	0.613	1.002	0.422–2.377	0.997
No	97 (51.3)	29 (15.3)	4 (2.1)					
Black nightshade								
Yes	20 (10.6)	10 (5.3)	0 (0.0)	2.484	0.289	1.218	0.434–3.417	0.708
No	117 (61.9)	36 (19.0)	6 (3.2)					
Mangoes								
Yes	68 (36.0)	27 (14.3)	2 (1.0)	1.934	0.380	1.191	0.531–2.668	0.671
No	69 (36.5)	19 (10.1)	4 (2.1)					
Guavas								
Yes	56 (29.6)	22 (11.6)	1 (0.5)	2.293	0.318	1.287	0.580–2.857	0.535
No	81 (42.9)	24 (12.7)	5 (2.7)					
Lime								
Yes	30 (15.9)	10 (5.3)	2 (1.0)	0.443	0.801	1.075	0.431–2.678	0.877
No	107 (56.6)	36 (19.0)	4 (2.1)					
Tamarind								
Yes	6 (3.2)	4 (2.1)	0 (0.0)	1.626	0.443	1.480	0.346–6.327	0.597
No	131 (69.3)	42 (22.2)	6 (3.2)					

*Pearson Chi-Square (χ2) test for associations. *Statistically significant χ2 association at α ≤ 0.05. OR odds ratio, 95% CI: 95% Confidence Interval. OR generated through ordinary logistic regress using PLUM test procedure

As shown in Table 9, during harvesting season, production of kidney beans was associated with a 62% reduction in the odds of children being severely food insecure (OR = 0.379, 95% CI = 0.190–0.754, *p* = 0.006). Children’s food security status was not comparable to production of; soya beans (*p* = 0.650), millet (*p* = 0.346), sorghum (*p* = 0.844), cassava (*p* = 0.604), sweet potatoes (*p* = 0.696),ground nuts (*p* = 0.412), green grams (*p* = 0.556), cow peas (*p* = 0.414), amaranth (*p* = 0.297), spider plant (*p* = 0.533), black night shade (*p* = 0.100), mangoes (*p* = 0.700), guavas (*p* = 0.334), lime (*p* = 0.102), and tamarind (*p* = 0.984).

**Table 9:** Associations between indigenous food production and children's food security status during harvesting season

Variables	Food Security Status			*χ* ^2^	*p*-value	OR	95% CI	*p*-value
	Food secure	Moderately food insecure	Severely food insecure					
	*n* (%)	*n* (%)	*n* (%)					
**Indigenous food production**								
Kidney Beans								
Yes	46 (23.8)	21 (10.9)	4 (2.0)	9.035	0.011*	0.379	0.190–0.754	0.006*
No	56 (29.0)	38 (19.7)	24 (12.4)					
Soya Beans								
Yes	3 (1.6)	2 (1.0)	0 (0.0)	0.922	0.631	0.620	0.079–4.883	0.650
No	99 (51.3)	57 (29.5)	28 (14.4)					
Millet								
Yes	1 (0.5)	5 (2.6)	1 (0.5)	5.888	0.053*	2.103	0.448–9.868	0.346
No	101 (52.3)	54 (28.0)	27 (14.0)					
Sorghum								
Yes	19 (9.8)	10 (5.2)	5 (2.6)	0.072	0.965	0.921	0.404–2.098	0.844
No	83 (43.0)	49 (25.4)	23 (11.9)					
Cassava								
Yes	29 (15.0)	22 (11.4)	6 (3.1)	2.581	0.275	1.226	0.568–2.644	0.604
No	73 (37.8)	37 (19.2)	22 (11.4)					
Sweet Potatoes								
Yes	29 (15.0)	17 (8.8)	6 (3.1)	0.613	0.736	0.865	0.418–1.789	0.696
No	73 (37.8)	42 (21.8)	22 (11.4)					
Ground Nuts								
Yes	35 (18.1)	27 (14.0)	8 (4.1)	3.111	0.211	1.333	0.671–2.647	0.412
No	67 (34.7)	32 (16.6)	20 (10.4)					
Green Grams								
Yes	9 (4.7)	7 (3.6)	3 (1.6)	0.398	0.819	1.377	0.475–3.995	0.556
No	93 (48.2)	52 (26.9)	25 (12.9)					
Cow Peas								
Yes	28 (14.5)	16 (8.3)	9 (4.7)	0.276	0.871	1.334	0.669–2.660	0.414
No	74 (38.3)	43 (22.3)	19 (9.8)					
Amaranth leaves								
Yes	32 (16.6)	16 (8.3)	6 (3.1)	1.153	0.562	0.679	0.328–1.405	0.297
No	70 (36.3)	43 (22.3)	22 (11.4)					
Spider Plant								
Yes	30 (15.5)	23 (11.9)	6 (3.1)	3.061	0.216	0.784	0.365–1.684	0.533
No	72 (37.3)	36 (18.7)	22 (11.4)					
Black nightshade								
Yes	13 (6.7)	12 (6.2)	5 (2.6)	1.711	0.425	2.142	0.865–5.302	0.100
No	89 (46.1)	47 (24.4)	23 (11.9)					
Mangoes								
Yes	54 (28.0)	33 (17.1)	10 (5.2)	3.339	0.188	1.147	0.570–2.308	0.700
No	48 (24.8)	26 (13.5)	18 (9.3)					
Guavas								
Yes	49 (25.4)	20 (10.4)	10 (5.2)	3.573	0.168	0.703	0.343–1.439	0.334
No	53 (27.4)	39 (20.2)	18 (9.3)					
Lime								
Yes	28 (14.5)	8 (4.2)	6 (3.1)	4.185	0.123	0.491	0.210–1.151	0.102
No	74 (38.3)	51 (26.4)	22 (11.4)					
Tamarind								
Yes	6 (3.1)	4 (2.1)	0 (0.0)	1.896	0.387	1.016	0.233–4.423	0.984
No	96 (49.7)	55 (28.5)	28 (14.5)					

*Pearson Chi-Square (χ2) test for associations. *Statistically significant χ2 association at α ≤ 0.05. OR odds ratio, 95% CI: 95% Confidence Interval. OR generated through ordinary logistic regress using PLUM test procedure

## Discussion

The study provides information on seasonality in associations between indigenous food production and food security status of mothers and children aged 12 to 36 months across two study seasons (planting and harvesting seasons). The projected reduction in food insecurity attributable to risks posed by climate change to mothers in Sub-Saharan Africa in indigenous communities is challenging, complex, and under-researched. A high proportion of lactating mothers globally are highly vulnerable to nutritional deficiency due to dietary monotony and food insecurity, which are noteworthy undernutrition determinants [25]. Mothers and children in low-resource regions are susceptible to adverse impacts of food insecurity due to climate change that derails overall food production [26]. In Fig. 1, severe food insecurity prevalence of mothers was 137 (71.0%) and 121 (62.7%) in planting and harvesting seasons, respectively. This compares to findings from a study on food insecurity and dietary diversity among lactating women in Nepal, which reported food insecurity prevalence of 54.0% [25]. Women encounter constraints in accessing an adequate and quality diet due to the low productivity of indigenous food [27]. Hence, ensuring sustained food security among societies who rely on indigenous foods as their primary food source depends on continuous access to sufficient traditional food resources in households, especially during short rain periods [25].

Seasonality in food production is typical in subsistence agricultural economies such as those of the study context; Seme. Deductively, mothers drawn from the present study setting; Seme, are vulnerable to substantial alterations throughout the year due to high food costs in the local market and crop production variability [28]. Participants observed that there was little focus on home-grown solution to food insecurity in the study context, which further exacerbate food insecurity situation; *“… the community rarely forges for locally available remedies to food insecurity such as adoption of traditional foods such as edible insects that are readily available*” (female participant). Participants further noted that residents from the study setting live in a constant state of worry attributable to inability to cater for their household’s dietary needs; “… *most household members are often forced to skip meals and give priority to vulnerable household members such as children”* (male participant). Diversified local-food production is crucial in meeting mothers’ dietary needs, alleviating malnutrition, and providing sustainable food security to attain year-round household security [29]. Different indigenous foods such as fruit trees are available, accessible, and affordable and confer known positive health outcomes [30]. Dried indigenous vegetables are a safe food source due to their shelf-life extension in meeting mothers’ satisfaction during the off-seasons [31]. Again, food security and production affiliation with preservation and processing are unique, as fermentation and drying techniques should be carried out during scarcity and abundance times [32]. The above mechanism ensures progressive and stable food availability throughout the year. However, the current study did not explore such mechanism, but instead focused on production aspect, which exhibited weak and no significant associations with food insecurity across both study seasons.

Tables 2 and 6 shows absence of significant associations between production of almost all the selected indigenous foods; grains, nuts and legumes, fruits, and vegetables and food security status. This compares to findings from a study on food insecurity intervention mechanisms in Guatemala’s indigenous agricultural communities, which observed absence of significant associations between production/sale of grains, nuts and legumes, other fruits, and vegetables and food security status; *p* = 0.912, 0.814, 0,734, and 0.304, respectively [33]. However, the Guatemalan study further assessed association between food items produced/sold within grocery stores and observed significant association between sale of eggs and dairy products and food security status; *p* = < 0.0001 and 0.004, respectively. The same study further observed existence of significant association between production (stocking at grocery stores) of only eggs and dairy products, which we did not focus on in our present study.

Table 7 reveal an association between sorghum production and children’s food security status in planting season (OR = 3.498, 95% CI = 1.454–8.418, *p* = 0.005). Similarly, Table 8 shows that kidney beans was associated with a 62% reduction in the odds of children being severely food insecure (OR = 0.379, 95% CI = 0.190–0.754, *p* = 0.006). These findings compare to those from a novel study done in Southern and Central Mali that used Simpson diversity index and multinomial logistics to assess crop diversification. This study reports that strategies promoting diversified crop production provide a viable means to resolving children’s undernutrition concerns that are often attributable to food insecurity [34]. The prospect of alleviating undernutrition attributable to food insecurity often rely on diversified food crop production as a means of supplementing other nutritional interventions. Diversified food crop production benefits household and children’s food security status [35]. Diversified food crop production is a dynamic and sustainable approach that strengthens resilience of children’s’ food security status [36]. These notions concur with World Bank’s report that indigenous crop diversification enhances cereals and staple foods in attaining self-sufficient food production [37].

Another study done in Bamako, Mali observed significant associations in the production of four indigenous food crops (sorghum, cereals, millet, and groundnuts) with children’s food security status; *p* = 0.013 [34]. In Table 8 and 7, respectively, production of kidney beans and sorghum demonstrated an association with children’s food security status in the present study. As such, findings from the above-mentioned Malian study affirm that mothers highly value sorghum production for children’s consumption due to its nutritional value. Absence of significant associations between production of most of the indigenous foods and children’s food security status in the current study could have been as a result of low production of these foods. In most instances, poor households adopt poor feeding options; feed their children with cheap caloric food due to high expenditure in acquiring food in case of low productivity [38]. Therefore, low indigenous food production reported in the present study may have limited the ability of these foods to influence children’s nutritional status; hence, food security status outcomes.

A study done in Northern Ghana reported a significant empirical link between indigenous food crop production and children’s nutrition status, which is a core indicator of food security status; *p* = < 0.0001 [39]. An empirical study comparing food security status of children from non-producer households reports that such children are often at a higher risk of being food insecure as evident by augmented wasting, stunting, and underweight rates [40]. From the Asian perspective, India has been identified as a famous nation with the highest rate of wasted children below five years; hence, the country may fail to meet the global 2025 nutrition target for wasting [41]. Agricultural production poses an influence on child nutrition as it influences child feeding practices [42]. Many times, agricultural vulnerability to global warming and climate change has been adversely influencing malnutrition among Indian children [43]. Relatedly, World Health Organization (WHO) reports that inappropriate meal consumption and dietary diversity related to limited food commodity production among children below five years result in inadequate nutritional status outcomes [44]. Therefore, adequate nutrition is essential for optimum health, growth, and child development.

Dietary diversity is a proxy indicator of sufficiency in nutrient density of food consumption in children because less meal frequency indicates inability to meet children’s energy requirements [45]. Production rate of indigenous food crops, cereals, and pulses determines the minimum acceptable diet, minimum meal frequency, and minimum dietary diversity [42]. This means that indigenous food production often exhibits significant associations with food security status. This was evident from findings from a study that found that production of cash crops negatively affected children’s diet and food security status [42]. Chronic stunting and malnutrition are often a result of inadequate micronutrient consumption caused by low food production [46]. Proper application and implementation of agricultural interventions stand a chance to improve children’s complementary feeding practices and nutritional (food security) status [47]. Thus, there is need to intensify global consensus on coordination mechanisms and multi-Sectoral approaches such as enhanced indigenous food production when addressing the debilitating malnutrition impacts amongst children [48].

## Conclusions

Severe food insecurity prevalence was higher amongst mothers during planting season when compared to harvesting season. On the contrary, severe food insecurity prevalence was higher amongst children during harvesting season when compared to the planting season. From these findings, we recommend that interventions to address food insecurity amongst mothers and children should consider variations in food insecurity prevalence amongst these two populations during planting and harvesting seasons. The study demonstrated no associations between production of selected indigenous foods and food security status of mothers. There was a weak association between indigenous food production and children’s food security status. The results imply that indigenous food production pose mixed outcomes with regards to food security status of mothers and children. It is on this basis that we recommend an intervention-based study that would be best suited in establishing existence of causal associations between indigenous food production and food security status of mothers and children. Nonetheless, the study limitation aligns with the fact that we did not assess for precise measures of indigenous foods produced by the participant’s households.

## Data Availability

As per the privacy and confidentiality provisions in the informed consent, the dataset generated and analysed for the study are not available publicly. However, data can be obtained from the corresponding author (kenkipton@gmail.com) upon reasonable request.

## Abbreviations

FANTA: Food and Nutrition Technical Assistance
FGD: Focused Group Discussions
HFIAS: Household Food Insecurity Access Scale
KIIs: Key Informant Interviews
PoU: Prevalence of Underweight
SSA: Sub-Saharan Africa
UNICEF: United Nations Children’s Fund
